# Draft Genome Sequence of *Lactobacillus plantarum* SF2A35B

**DOI:** 10.1128/genomeA.01638-15

**Published:** 2016-02-25

**Authors:** Peter A. Bron, I-Chiao Lee, Lennart Backus, Sacha A. F. T. van Hijum, Michiel Wels, Michiel Kleerebezem

**Affiliations:** a TI Food and Nutrition, Wageningen, The Netherlands; b NIZO food research, Ede, The Netherlands; c Host Microbe Interactomics Group, Wageningen University & Research Centre, Wageningen, The Netherlands; d Centre for Molecular and Biomolecular Informatics, Radboud Institute for Molecular Life Sciences, Radboudumc, Nijmegen, The Netherlands

## Abstract

The lactic acid bacterium *Lactobacillus plantarum* is intensively studied as a model probiotic species. Here, we present the draft genome sequence of the exopolysaccharide-producing strain SF2A35B.

## GENOME ANNOUNCEMENT

Large sets of potentially probiotic *Lactobacillus plantarum* strains have been characterized for their capacity to induce cytokine secretion by immune cells. One such study revealed that the application of *L. plantarum* strain SF2A35B to dendritic cells did not trigger the secretion of any cytokines, suggesting that this strain is hardly recognized by the immune system (1, 2). Notably, this strain produces significant amounts of exopolysaccharide (EPS), which has been proposed as a shielding molecule in this species (3, 4).

Ten milliliters of exponentially growing cells (optical density at 600 nm [OD_600_], 1.0) were harvested by centrifugation and resuspended in 0.5 ml of THMS (30 mM Tris [pH 8.0], 3 mM MgCl_2_ in 25% sucrose) containing 50 mg/ml lysozyme. This mixture was incubated at 37°C for 2 h, and cells were harvested by centrifugation and resuspension of cell pellets was done in 0.5 ml TE (10 mM Tris, 1 mM EDTA [pH 7.4]) containing 10 µg/ml RNase. Thirty microliters of a 10% (wt/vol) sodium dodecyl sulfate (SDS) solution was added, and the resulting mixture was incubated for 15 min at 37°C. Subsequently, 10 µl of a solution containing 20 mg/ml proteinase K was added, which was incubated for 15 min at 65°C. Phenol-chloroform extractions were repeatedly performed until the water phase was clear, and residual traces of phenol were removed from the water phase by chloroform extraction. Total DNA was precipitated from the water phase by addition of 1 volume of isopropanol. The DNA was pelleted by centrifugation and washed once with 70% ethanol, vacuum dried, and dissolved overnight in 1 ml of TE at 4°C (5).

The whole-genome sequencing was performed at GATC Biotech (Konstanz, Germany), and paired-end 50-bp libraries were made and sequenced using an Illumina HiSeq 2000. The raw sequence reads of each of the genomes were assembled *de novo* using the Ray assembler (6). The *L. plantarum* SF2A35B genome is estimated to be 3.21 Mb, is assembled into 113 contigs, and has a G+C content of 45.08%. Annotation was performed by automated annotation of the scaffold sequences using the RAST server (7). In total, 3,074 genes were predicted. Three clusters predicted to be involved in exopolysaccharide production were identified. One cluster harbored 5 unique genes compared to the *L. plantarum* type strain WCFS1 (8), which might encode functions involved in the ropy phenotype of this strain.

### Nucleotide sequence accession numbers.

This whole-genome shotgun project has been deposited at DDBJ/EMBL/GenBank under the accession no. LMVD00000000. The version described in this paper is version LMVD01000000.
